# Overexpression of *SrDXS1* and *SrKAH* enhances steviol glycosides content in transgenic Stevia plants

**DOI:** 10.1186/s12870-018-1600-2

**Published:** 2019-01-03

**Authors:** Junshi Zheng, Yan Zhuang, Hui-Zhu Mao, In-Cheol Jang

**Affiliations:** 1 Temasek Life Sciences Laboratory, 1 Research Link, National University of Singapore, Singapore, 117604 Singapore; 2 Department of Biological Sciences, National University of Singapore, Singapore, 117558 Singapore

**Keywords:** *1-deoxy-d-xylulose-5-phosphate synthase 1*, *Kaurenoic acid hydroxylase*, Metabolic engineering, Stevia transformation, Steviol glycosides, Transgenic Stevia

## Abstract

**Background:**

*Stevia rebaudiana* produces sweet-tasting steviol glycosides (SGs) in its leaves which can be used as natural sweeteners. Metabolic engineering of Stevia would offer an alternative approach to conventional breeding for enhanced production of SGs. However, an effective protocol for Stevia transformation is lacking.

**Results:**

Here, we present an efficient and reproducible method for *Agrobacterium*-mediated transformation of Stevia. In our attempts to produce transgenic Stevia plants, we found that prolonged dark incubation is critical for increasing shoot regeneration. Etiolated shoots regenerated in the dark also facilitated subsequent visual selection of transformants by green fluorescent protein during Stevia transformation. Using this newly established transformation method, we overexpressed the Stevia *1-deoxy-d-xylulose-5-phosphate synthase 1* (*SrDXS1*) and *kaurenoic acid hydroxylase* (*SrKAH*), both of which are required for SGs biosynthesis. Compared to control plants, the total SGs content in *SrDXS1-* and *SrKAH-*overexpressing transgenic lines were enhanced by up to 42–54% and 67–88%, respectively, showing a positive correlation with the expression levels of *SrDXS1* and *SrKAH*. Furthermore, their overexpression did not stunt the growth and development of the transgenic Stevia plants.

**Conclusion:**

This study represents a successful case of genetic manipulation of SGs biosynthetic pathway in Stevia and also demonstrates the potential of metabolic engineering towards producing Stevia with improved SGs yield.

**Electronic supplementary material:**

The online version of this article (10.1186/s12870-018-1600-2) contains supplementary material, which is available to authorized users.

## Background

*Stevia rebaudiana* is a perennial shrub that belongs to the Asteraceae family. It produces steviol glycosides (SGs) that range from 150 to 300 times as sweet as sucrose, making it unique among plants [1]. SGs are mainly accumulated in the leaves of Stevia, accounting for around 4–20% of leaf dry weight [2]. In Paraguay where Stevia is native to, people have long been using it to sweeten their teas and medicine [3]. In recent times, the value of Stevia leaf extracts or specific SG, like Rebaudioside A (Reb A), as a zero calorie natural sweetener has also gained recognition beyond its native country, leading to the introduction of Stevia as a commercial crop in many other countries [1].

SGs are a group of diterpenoids with varying levels of sweetness depending on the different number and types of sugar moieties (glucose, rhamnose, or xylose) substituted on its aglycone, steviol [4]. Steviol is synthesized through the methylerythritol phosphate (MEP) pathway in the chloroplast [5]. The first step in the MEP pathway involves the condensation of pyruvate and d-glyceraldehyde-3-phosphate into 1-deoxy-d-xylulose-5-phosphate (DXP) by DXP synthase (DXS) [6]. After six more steps of conversion, the final enzyme 4-hydroxy-3-methylbut-2-enyl pyrophosphate reductase converts (*E*)-4-hydroxy-3-methylbut-2-enyl pyrophosphate into isopentenyl pyrophosphate (IPP) and dimethylallyl pyrophosphate (DMAPP), which are the basic five-carbon precursors for the formation of all terpenoids. For the production of SGs and other diterpenoids, two intermediates, IPP and DMAPP, undergo consecutive condensation to form C_20_ geranylgeranyl pyrophosphate (GGPP). GGPP is then further cyclized to (−)-kaurene and subsequently oxidized to kaurenoic acid [7, 8]. All steps leading to the formation of kaurenoic acid are also common to gibberellic acid (GA) biosynthesis [9]. However, the hydroxylation of kaurenoic acid at C-13 position by kaurenoic acid hydroxylase (KAH) diverts it towards SG biosynthesis [9]. Finally, UDP-glycosyltransferases (UGTs) add sugar moieties at the C-13 or C-19 position of steviol to produce a variety of SGs [10].

Many Stevia genes uncovered from the next-generation sequencing are now publicly available [11, 12]. However, a reliable Stevia transformation technology remains to be developed for the functional genomics of Stevia and the generation of new Stevia with improved traits such as greater sweetness and resistance towards pests and diseases. Although *Agrobacterium*-mediated transformation of Stevia using β-glucuronidase (GUS) reporter gene was introduced [13], no further transgenic Stevia has been reported so far, which may result from the absence of a reliable transformation method. Tobacco plants have been routinely transformed using *Agrobacterium* and its protocol could be conveniently adapted to plants of Solanaceae family [14–17]. However, the transformation of other important crops such as soybean and corn required further optimization of their specific regeneration strategies [18]. For Stevia, although there are a few protocols describing shoot regeneration from leaf explants, there has been a lack of consensus on the conditions used [19–21]. Therefore, the development of a new and efficient method for regeneration and genetic transformation of Stevia would be required for a broad range of biotechnological applications as well as functional genomic studies of Stevia.

Here we describe an efficient and reliable method for the *Agrobacterium*-mediated transformation of Stevia and demonstrate that using this method, we could obtain transgenic Stevia plants expressing the green fluorescent protein (GFP) from leaf explants. As a further demonstration of the efficacy of our transformation method, we transformed *SrDXS1* and *SrKAH* into Stevia. By *SrDXS1* overexpression, we successfully increased the total SGs content in the transgenic lines compared to control by up to 54%. Moreover, *SrKAH* overexpression in Stevia resulted in an even higher increase in total SGs content of up to 88%. Despite the increase in SGs content, the normal growth and development of Stevia were not compromised for both *SrDXS1*- and *SrKAH*-overexpression lines.

## Results

### Callus induction and shoot regeneration from Stevia leaf explants

**Fig. 1:**
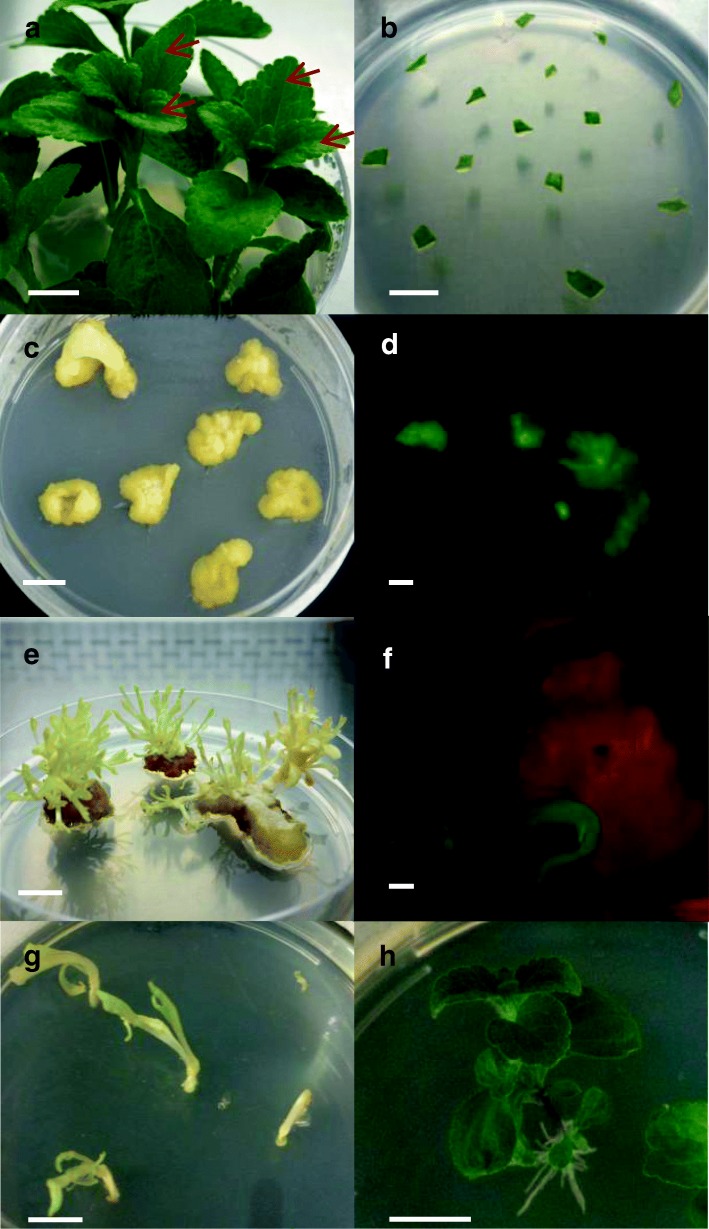
*Agrobacterium*-mediated transformation of Stevia using Condition F. **a** The red arrows indicate the second and third leaves that were used as the explant source. **b** Leaf explants on CCM. **c** Induced callus on CIM. **d** Transformed callus showing GFP fluorescence under a fluorescence stereomicroscope. **e** Shoots regenerated from calli on SIM. **f** Shoot regenerated from transformed calli showing GFP fluorescence under a fluorescence stereomicroscope. **g** Regenerated shoots on RM. **h** Rooting of regenerated shoots on RM. Scale bars = 1 cm for (**a**-**c**, **e**, **g** and **h**); 1 mm for (**d)** and (**f**). CCM, co-cultivation media; CIM, callus induction media; SIM, shoot induction media; RM, rooting media

Plant transformation involves a few major steps namely, co-cultivation, callus induction, shoot regeneration and root regeneration, but all these steps require optimization to suit individual plants. To establish a standard transformation method for Stevia, we investigated the effects of different hormone combinations on callus induction and shoot regeneration by modifying existing procedures for tobacco transformation (Table 1) [15]. We chose the second and third leaves of in vitro cultured Stevia plants as the explant source (Fig. 1a).

**Table 1:** Cytokinin and auxin combinations tested for callus induction and shoot regeneration from Stevia leaf explants

Condition	CCM (mg/L)	CIM (mg/L)	SIM (mg/L)
A	–	BA 1 + NAA 2	BA 1 + NAA 2
B	–	BA 1 + NAA 0.5	BA 1 + NAA 0.5
C	–	BA 1 + IAA 2	BA 1 + IAA 2
D	–	BA 1 + IAA 0.5	BA 1 + IAA 0.5
E	2,4-D 0.25	BA 1 + IAA 0.5	BA 1 + IAA 0.5
F	2,4-D 0.25	BA 1 + IAA 0.5	BA 2 + IAA 0.25
F-light	2,4-D 0.25	BA 1 + IAA 0.5 ^a^	BA 2 + IAA 0.25^a^

*CCM* co-cultivation media, *CIM* callus induction media, *SIM* shoot induction media, *BA* 6-benzylaminopurine, *NAA* 1-naphthaleneacetic acid, *IAA* 3-indoleacetic acid; 2,4-D, 2,4-dichlorophenoxyacetic acid

^a^ Explants were incubated under light with 16 h L/ 8 h D photoperiod

Plant growth regulators most frequently supplemented for shoot regeneration from Stevia leaf explants include 6-benzylaminopurine (BA) as the cytokinin and 1-naphthaleneacetic acid (NAA), or 3-indoleacetic acid (IAA) as the auxin [19–21]. When explants were placed on BA with either NAA or IAA under long day photoperiod (LD, 16 h Light/ 8 h Dark), calli were induced on both media but with a different appearance (Additional file 1: Figure S1a, b). Shoot regeneration could also be observed from the calli on the BA + IAA media after 6 weeks but its frequency would be insufficient for successful transformation (Additional file 1: Figure S1b). It has been shown that prolonged dark incubation promotes somatic embryogenesis from callus cultures of Stevia [22]. Interestingly, we found drastic improvements in shoot regeneration from calli induced in the dark (Additional file 1: Figure S1c). Therefore, we subsequently incubated the explants under darkness during callus induction and shoot regeneration.

To compare the efficiency of BA with IAA or NAA on callus induction and shoot regeneration, four combinations (Conditions A-D in Table 1) with different concentrations of NAA or IAA were designed. The difference in callus induction rates on four different callus induction media (CIM; Conditions A-D in Table 1) was not observed to be statistically significant (*P-*value: 0.099; Table 2). However, calli on CIM containing NAA (Conditions A and B) appeared friable while those on media containing IAA appeared compact (Conditions C and D; Table 2). Subsequently, calli maintained on NAA (Conditions A and B) had lower shoot regeneration rates than those on IAA (Conditions C and D; Table 2). Furthermore, we found that a higher BA to IAA ratio (Condition D) was more efficient for promoting shoot regeneration (Table 2).

**Table 2:** Callus induction and shoot regeneration rates under the different cytokinin and auxin combinations listed in Table 1

Condition	Explants with callus formation (%)	Callus condition	Explants with regeneration (%)	Shoot Condition
A	87.4 ± 2.5	Friable	5.0 ± 1.4	+
B	99.2 ± 0.8	Friable	22.8 ± 2.6	+ +
C	89.1 ± 5.1	Compact	29.4 ± 2.9	+ + + + +
D	98.3 ± 0.8	Compact	65.8 ± 3.6	+ + + +
E	95.0 ± 3.8	Compact	53.3 ± 5.1	+ + + + +
F	96.7 ± 3.3	Compact	53.3 ± 5.8	+ + + + +
F-light	95.8 ± 1.7	Compact	29.5 ± 7.7	+ + + +

Values are mean ± SE of technical triplicates with *n* = 40

The shoot condition was scored based on their appearance (+: Most shoots appear watery, browning or deformed, + + + + +: Most shoots appear strong and healthy)

2,4-dichlorophenoxyacetic acid (2,4-D) is commonly used for the dedifferentiation of somatic cells [23]. Therefore, to further enhance regeneration rates under Condition D, we designed Condition E with an additional 3 d incubation on 0.25 mg/L 2,4-D (Table 1), which can also be used as the co-cultivation media (CCM) for *Agrobacterium*-mediated transformation. Although regeneration rates for Conditions E were similar to Condition D, the regenerated shoots were healthier (Table 2 and Additional file 2: Figure S2a, b).

In general, a higher cytokinin to auxin ratio promotes shoot formation [24]. We further optimized Condition E by doubling the cytokinin concentration of the shoot induction media (SIM) to 2 mg/L and reducing the auxin concentration from 0.5 mg/L to 0.25 mg/L to form Condition F (Table 1). Under Condition F, rates for callus formation and shoot regeneration as well as the shoot condition were comparable to those under Condition E (Table 2), but the number of regenerated shoots per callus clump seemed to be higher (Additional file 2: Figure S2c). Next, we tested Condition F simultaneously under LD condition after the explants were transferred onto CIM (Condition F-light; Table 1) to verify the enhancement of shoot regeneration in the dark. Certainly, the percentage of explants with regenerated shoots was 1.8 times higher under Condition F (Table 2), confirming that dark incubation greatly promotes shoot regeneration. Therefore, we subsequently applied Condition F for Stevia transformation.

### Stevia transformation

To investigate the feasibility of adapting condition F for transformation, we co-cultivated Stevia leaf explants on the CCM media containing acetosyringone with *Agrobacterium* harboring the pK7WG2D vector [25], which contains a *neomycin phosphotransferase* (*nptII*) gene and an enhanced GFP gene fused to an endoplasmic reticulum targeting signal (*EgfpER*) to allow concurrent selection (Fig. 1b). Figure 1 outlines the overall procedures for *Agrobacterium*-mediated transformation of Stevia. The appearance of the calli and regenerated shoots on media are shown in Fig. 1c and e, respectively. Incubation in the dark resulted in their etiolated appearance. GFP signals from transgenic calli or regenerated shoots were monitored and selected under a fluorescence stereomicroscope (Fig. 1d, f). With reduced autofluorescence from chlorophyll, GFP signals could easily be visualized. For rooting, transgenic shoots were transferred onto rooting media (RM) and exposed to light for approximately 1 month (Fig. 1g, h). Using this approach, we were able to efficiently produce transgenic Stevia plants expressing GFP.

### Transformation of Stevia with SrDXS1 and SrKAH

DXS has been reported to play a rate-limiting role in the MEP pathway [26–28], while Stevia KAH acts on kaurenoic acid as the committed step to SGs biosynthesis [9]. Thus, we hypothesized that their overexpression would lead to an increase in the flux towards SGs production.

**Fig. 2:**
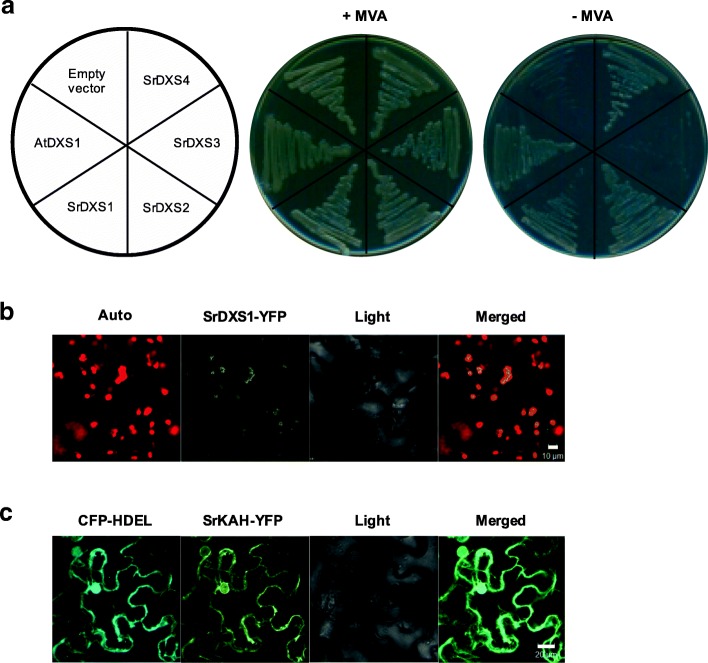
Characterization of *SrDXSs* and *SrKAH*. **a** Complementation assay of Stevia DXSs using *E. coli* DXS deficient mutant (*dxs*^−^). Transformed cells were grown on LB plates containing either with 0.5 mM mevalonate (+ MVA) or without mevalonate (− MVA). *E. coli dxs*^*−*^ with pDEST17 (empty vector) and *AtDXS1* served as negative and positive controls, respectively. **b** Subcellular localization of SrDXS1. Auto, chlorophyll autofluorescence; Light, light microscope image; Merged, merged image between Auto and YFP channels. Scale bar = 10 μm. **c** Subcellular localization of SrKAH. Co-expression of SrKAH-YFP with CFP-HDEL in. Light, light microscope image; Merged, merged image between CFP and YFP channels. Scale bar = 20 μm

Four Stevia *DXS* homologs (*SrDXS1–4*) were identified from the RNA-seq data of Stevia leaves [12]. To investigate if all four SrDXSs were functionally active, we carried out a complementation assay using a *dxs*-deficient *Escherichia coli*. Figure 2a shows that *dxs*^−^ *E. coli* transformed with all *SrDXSs* except *SrDXS3* were able to grow on selection media, similar to the Arabidopsis *DXS1* (*AtDXS1*) positive control, indicating their functionality. Among the 4 *SrDXS* homologs, only *SrDXS1* was suggested to be involved in SG biosynthesis based on the correlation between its expression pattern and the site of SGs biosynthesis [12]. Transient expression of the yellow fluorescent protein (YFP)-fused SrDXS1 in *Nicotiana benthamiana* leaves showed that it localizes to the chloroplast (Fig. 2b). Therefore, we selected *SrDXS1* for Stevia transformation.

Unlike SrDXS1, the activity of SrKAH in converting kaurenoic acid to steviol has previously been demonstrated in *E. coli* [29]. Additionally, its overexpression in Arabidopsis had led to the production of steviol that was otherwise not detected [30]. Being a cytochrome P450 enzyme, SrKAH is expected to be localized to the endoplasmic reticulum (ER) similar to that of kaurene oxidase which acts upstream of it [9]. We confirmed this by transiently co-expressing YFP-fused SrKAH and cyan fluorescent protein (CFP)-fused HDEL, an ER marker, in *Nicotiana benthamiana* leaves. Figure 2c shows the co-localization of SrKAH-YFP with CFP-HDEL, demonstrating that SrKAH indeed localizes to the ER.

**Fig. 3:**
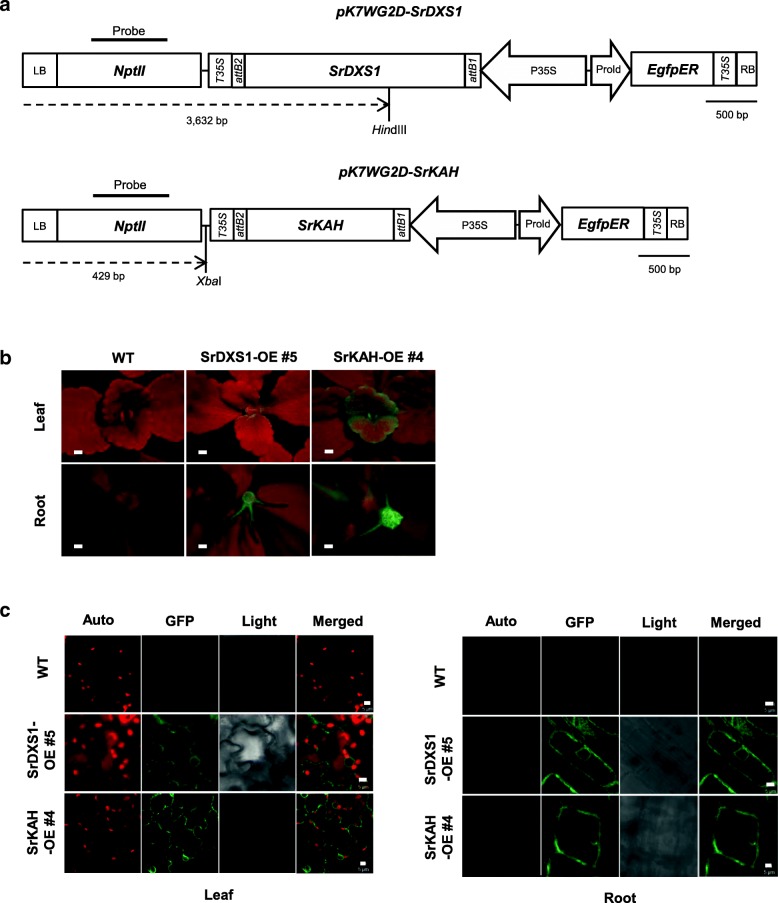
Identification of transgenic Stevia plants overexpressing *SrDXS1* (SrDXS1-OE) or *SrKAH* (SrKAH-OE). **a** Schematic maps of T-DNA region of pK7WG2D-SrDXS1 and pK7WG2D-SrKAH used for Stevia transformation. LB, left border; *nptII*, *neomycin phosphotransferase* marker gene under the terminator and promoter of nopaline synthase gene; *T35S* and P35S, terminator and promoter of the cauliflower mosaic virus gene respectively; *attB2* and *attB1*, gene recombination sites; *SrDXS1*, Stevia *1-deoxy-d-xylulose-5-phosphate synthase 1*; *SrKAH*, Stevia *kaurenoic acid hydroxylase* gene; *EgfpER,* enhanced green-fluorescent protein gene fused to endoplasmic reticulum targeting signal; ProlD, rol root loci D promoter; *Xba*I and *Hin*dIII, sites digested by *Xba*I and *Hin*dIII, respectively, for Southern blot analysis; Probe, probe used for Southern blot analysis. **b** Images of GFP signals from leaves and roots of representative SrDXS1-OE #6 or SrKAH-OE #4 under a fluorescence stereomicroscope. WT, wild-type. Scale bar = 1 mm. **c** Confocal images of the leaf underside and roots of WT, representative SrDXS1-OE #6 or SrKAH-OE #4. Auto, chlorophyll autofluorescence; GFP, GFP channel image; Light, light microscope image; Merged, merged image between Auto and GFP channels. Scale bar = 5 μm

Next, we cloned the full-length open reading frame (ORFs) of *SrDXS1* and *SrKAH* into pK7WG2D under the control of the cauliflower mosaic virus (CaMV 35S) promoter for Stevia transformation (Fig. 3a). Using our transformation protocol, we produced 13 and 9 lines of transgenic Stevia plants overexpressing *SrDXS1* (SrDXS1-OE) and *SrKAH* (SrKAH-OE), respectively. Because of the GFP visual marker, we were able to efficiently select the transgenic Stevia plants emitting GFP signals from leaf and root tissues of SrDXS1-OE and SrKAH-OE lines under a fluorescence stereomicroscope and a confocal laser scanning microscope (CLSM; Fig. 3b, c). GFP expressions in leaves of each transgenic Stevia lines were also confirmed by immunoblot analysis (Additional file 3: Figure S3).

### Analysis of transgenic Stevia lines

**Fig. 4:**
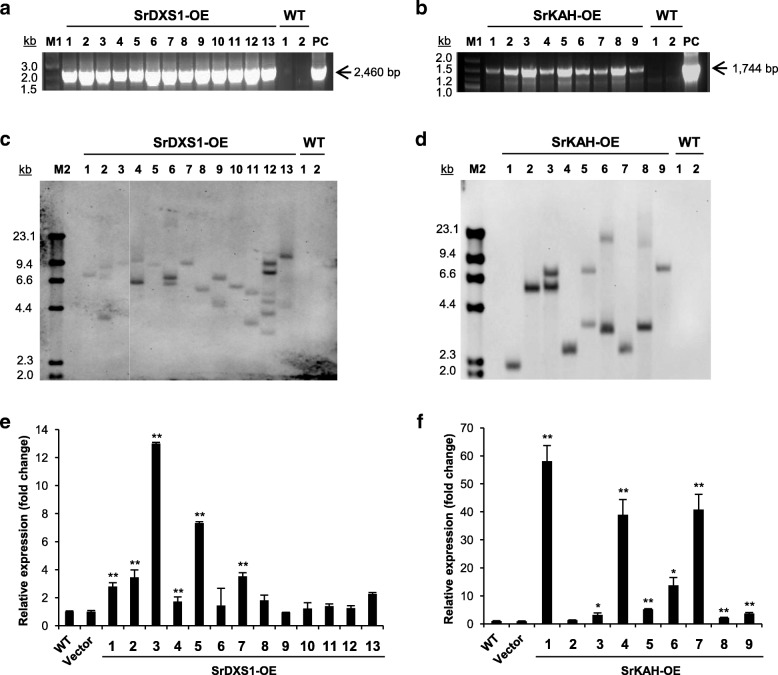
Genomic and expression analysis of transgenic Stevia plants overexpressing *SrDXS1* (SrDXS1-OE) or *SrKAH* (SrKAH-OE). **a** and **b** *SrDXS1* (**a**) or *SrKAH* (**b**) amplified from the gDNA of each transgenic Stevia lines. M1, 2-Log DNA ladder. PC, positive control amplified from the respective vector constructs. **c** and **d** Southern blot analysis of SrDXS1-OE (**c**) or SrKAH-OE lines (**d**). WT, wild-type. M2, DIG-labelled DNA molecular weight marker II. **e** and **f** Relative fold change in *SrDXS1* (**e**) and *SrKAH* (**f**) transcript levels among the transgenic Stevia lines overexpressing *SrDXS1* (SrDXS1-OE) and *SrKAH* (SrKAH-OE), respectively. Expression levels of both genes were normalized to that of *actin* and compared to that of wild-type (WT). The values are expressed as mean ± SE (*n* = 3). Student’s *t*-test was used for the analysis of statistical significance (*: *p* < 0.05, **: *p* < 0.01)

To verify if exogenous *SrDXS1* or *SrKAH* was integrated into the Stevia genome*,* genomic PCR analysis of the transgene from each transgenic line was performed. Genomic DNA amplification corresponding to the expected size of each transgene was observed for all the SrDXS1-OE or SrKAH-OE lines and the respective positive control lanes, but not for wild-type (WT; Fig. 4a, b).

After confirming the existence of full-length ORFs of each transgene in transgenic Stevia plants, we performed digoxygenin (DIG)-based Southern blot analysis to determine the number of transgene integration sites for each line with *nptII*-specific probe (Fig. 3a). Figure 4c and d show that all SrDXS1-OE and SrKAH-OE lines contained one or more transgene (*nptII*) integration site, demonstrating stable transgene integration into the Stevia genome. No bands were detected in the WT lanes.

Then, we analyzed the expression levels of *SrDXS1* and *SrKAH* in SrDXS1-OE and SrKAH-OE lines, respectively. Figure 4e shows up to 13-fold increase in the expression levels of *SrDXS1* among the transgenic lines compared to control. However, the expression levels of *SrDXS1* in SrDXS1-OE lines did not correlate with the number of transgene integration sites. Among the top 5 SrDXS1-OE lines, four of them had a single transgene integration site (Fig. 4c, e). For further analysis, we chose three lines, SrDXS1-OE #1, #3 and #5, each having one transgene integration site but different levels of *SrDXS1* overexpression.

Among SrKAH-OE lines that contained single transgene integration site, lines #1, #4 and #7 showed around 40–60 fold higher expression of *SrKAH* compared to that of WT while line #2 did not show *SrKAH* overexpression, and line #9 only had a small increase of around 4-fold (Fig. 4f). For further analysis of the effects of *SrKAH* overexpression, we selected lines #1, #4, and #9 with varying expression levels, and included line #2 as an internal control.

### Steviol glycosides (SGs) content increased in transgenic Stevia plants

It is known that Stevia is a self-incompatible plant and its self-pollination results in sterile seed set [31]. Under our environmental conditions, we were also unsuccessful in harvesting viable transgenic T1 seeds. Therefore, we propagated the in vitro transgenic lines by cutting method and monitored the GFP signals emitted. Transgenic Stevia plants showing GFP expression in whole tissues were transferred into the soil for hardening and grown in the greenhouse for 3 weeks before analysis. Using this method, we were able to maintain each transgenic line for further analysis and obtain reproducible results.

**Fig. 5:**
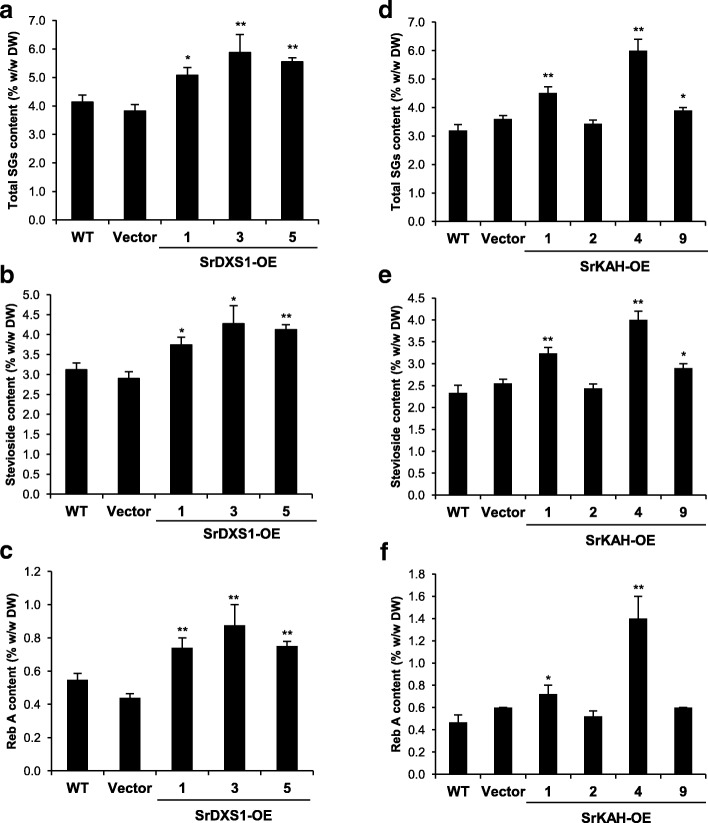
Analysis of steviol glycosides (SGs) content in transgenic Stevia plants. **a-f** Total SGs (**a** and **d**), stevioside (**b** and **e**) and Reb A (**c** and **f**) content in the transgenic Stevia lines overexpressing either *SrDXS1* (SrDXS1-OE) or *SrKAH* (SrKAH-OE). Data are presented as mean ± SE. Statistical analysis was carried out using Student’s *t*-test relative to wild-type (WT) (*n* = 5, *: *p* < 0.05, **: *p* < 0.01)

To investigate the effect of *SrDXS1* or *SrKAH* overexpression on SGs production, we analyzed the leaf extracts of the transgenic lines. As leaf SGs content can differ according to their nodal position, leaves from the same position of each line were harvested. Each SG peak was identified by comparing their retention time with that of their authentic standards (Additional file 4: Figure S4). By summing up the concentration of the top 4 most abundant SGs (stevioside, Reb A, Reb C and dulcoside A) in each of the SrDXS1-OE lines, we found an increase in SGs content in the transgenic lines as compared to the controls (Fig. 5a). The total SGs content was the highest in SrDXS1-OE line #3 at 5.9% (*w*/w dry weight, DW), followed by 5.6% (w/w DW) in line #5 and lastly 5.1% (w/w DW) in line #1 (Fig. 5a), in agreement with their relative *SrDXS1* expression levels (Fig. 4e). These total SGs content in the transgenic lines represent an increase of between 33-54% and 23–42% compared to the 3.8% (*w*/w DW) and 4.1% (w/w DW) total SGs content in the vector-only control line and WT, respectively (Fig. 5a). Stevioside, which is the most abundant SG in Stevia, had concentrations of between 3.7–4.3% (w/w DW) in the overexpression lines, increasing up to 20–47% compared to controls (Fig. 5b). Similar patterns of SGs increase for Reb A, Reb C and dulcoside A were found in SrDXS1-OE lines (Fig. 5c and Additional file 5: Figure S5a). Furthermore, in SrDXS1-OE line #9 where *SrDXS1* transcript levels were comparable to controls, the stevioside and Reb A contents were also similar (Fig. 4e and Additional file 6: Figure S6). These results suggest that the overexpression of *SrDXS1* in Stevia leads to a proportional increase in each SG.

In the SrKAH-OE lines, the total amount of SGs was able to reach up to 88% higher than that of WT (Fig. 5d). Corresponding to their expression levels, SrKAH-OE lines #1 and #4 accumulated the highest total amount of SGs at 4.5% (w/w DW) and 6% (w/w DW), respectively (Figs. 4f and 5d). On the other hand, SrKAH-OE #9 with only a four-fold increase in *SrKAH* transcript had total SGs content of 3.9% (w/w DW), indicating a moderate increase of 8–22% from the controls (Figs. 4f and 5d). SrKAH-OE line #2, an internal control line that shows similar expression levels of *SrKAH* with WT, did not contain higher total SGs content, confirming that elevated *SrKAH* transcript levels resulted in higher SGs in transgenic Stevia plants (Fig. 5d). Taking a closer inspection at the individual SGs, stevioside was present in concentrations of up to 4% (w/w DW) among the overexpression lines, which was an increase of 57–71% compared to controls (Fig. 5e). For Reb A, a 133–200% increase compared to controls was observed in SrKAH-OE #4 (Fig. 5f). Apart from stevioside and Reb A, statistically significant increases of Reb C and dulcoside A content were also found in the two *SrKAH* high expressers, SrKAH-OE lines #1 and #4, with patterns of increase similar to that of the total SGs content (Additional file 5: Figure S5b).

### Phenotype of transgenic Stevia plants

**Fig. 6:**
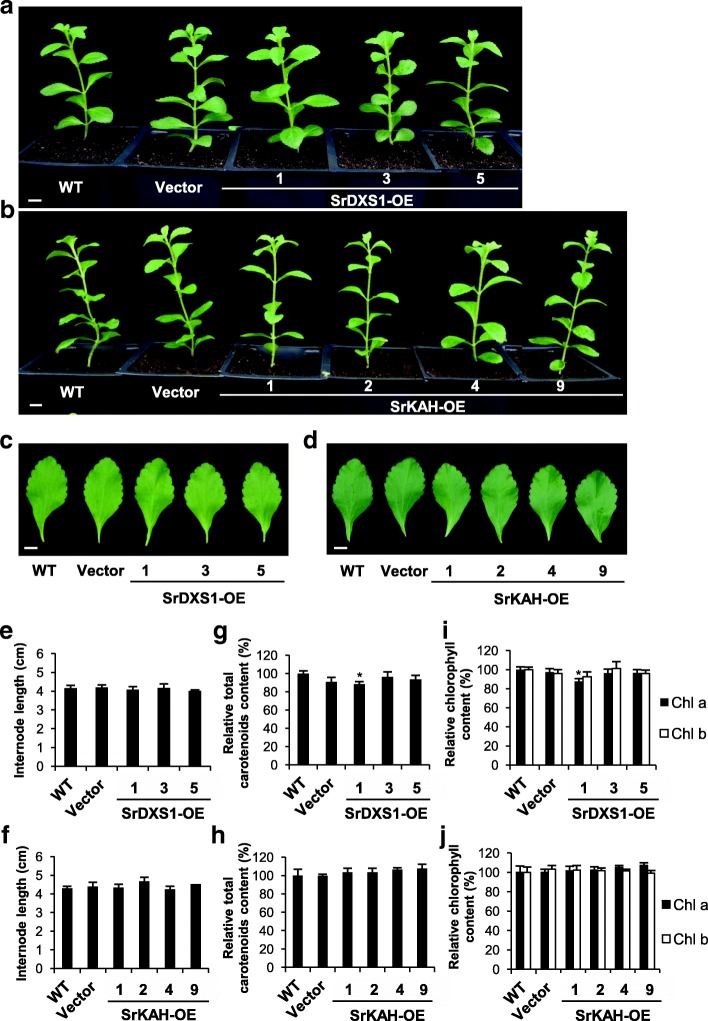
Phenotypic analysis of transgenic Stevia plants. **a** and **b** Representative transgenic Stevia plants overexpressing *SrDXS1* (SrDXS1-OE) (**a**) or *SrKAH* (SrKAH-OE) (**b**) one week after hardening in the soil. **c** and **d** Representative leaf harvested from third node position of SrDXS1-OE lines (**c**) or SrKAH-OE lines (**d**) one month after being transferred to the soil. **e** and **f** Average length of the third and fourth internodes in the SrDXS1-OE (**e**) or SrKAH-OE (**f**) lines one month after being transferred to the soil. Wild-type (WT) and vector-only line were included as a control. Scale bar = 1 cm. **g-j** Relative chlorophylls content and total carotenoids content in the transgenic Stevia plants overexpressing *SrDXS1* (SrDXS1-OE) (**g** and **i**) or *SrKAH* (SrKAH-OE) compared to wild-type (WT) (**h** and **j**). All measurements are expressed as mean ± SE (*n* = 5) and statistical analysis was carried out using Student’s *t*-test relative to wild-type (WT) for SrKAH-OE lines (*n* = 5, *: *p* < 0.05)

To determine if the overexpression of *SrDXS1* and *SrKAH* would result in other changes in the Stevia plant, we examined their phenotype. SrDXS1-OE lines did not display any morphological differences from controls. The height of the plants, size of the leaves and the internode length among the 2 month-old Stevia plants were comparable (Fig. 6a, c, e). Figure 6b, d and f show that SrKAH-OE lines also did not exhibit any obvious differences in growth. The leaf size and color and internode length were indistinguishable from the controls.

Apart from morphology, we also determined the relative concentration of chlorophyll *a*, chlorophyll *b* and total carotenoids because these compounds are also derived from the MEP pathway [6]. Figure 6g-j shows that except for SrDXS1-OE #1, there were no significant changes in chlorophylls and carotenoids content in both SrDXS1-OE and SrKAH-OE lines when compared to WT. Furthermore, compared to Vector-only control, chlorophyll *a* and total carotenoid content in SrDXS1-OE #1 were also not significantly different. Additionally, we measured the concentration of a few monoterpenes that were present in the Stevia leaf tissues since monoterpenes can also be synthesized from the MEP pathway [12]. Using gas chromatography–mass spectrometry (GC-MS) analysis, the relative amount of linalool, α-pinene and β-pinene were determined (Additional file 7: Figure S7). There were no statistically significant changes to the amount of monoterpenes in the leaves of SrDXS1-OE lines compared to those of controls. Hence, our results show that both *SrDXS1* and *SrKAH* overexpression could increase SGs content in transgenic Stevia without changing the abundance of other metabolites or having any detrimental effects on their growth and development.

## Discussion

Since the whole transcriptome of Stevia has been sequenced [11, 12], the transformation of Stevia is indispensable not only in functional genomics for elucidating crucial genes such as those involved in SGs biosynthesis and stress response, but also for metabolic engineering to fulfill commercial interests in producing SGs more efficiently. Here, we optimized conditions for shoot regeneration from Stevia leaf explants and adapted it for the Stevia transformation. Among the different regeneration conditions tested, Condition F (CCM: 0.25 mg/L 2,4-D, CIM: 1 mg/L BA + 0.5 mg/L IAA and SIM: 2 mg/L BA + 0.25 mg/L IAA) with incubation in continuous darkness was the most ideal as approximately 53% of the starting explants have healthy regenerated shoots (Table 2). Even though Khan et al. [13] previously reported regeneration frequency of nearly 90%, our attempts to replicate their condition which is equivalent to the Condition A could only achieve a regeneration rate of 5% (Table 2).

While optimizing for shoot regeneration, we observed that a prolonged dark incubation could improve significantly the rate of shoot regeneration (Table 2). Similar findings have been reported in other plants such as rice and citrus [32, 33]. It has been suggested that increased reactive oxygen species (ROS) levels during light exposure inhibit shoot regeneration [34, 35]. Thus, the low shoot regeneration rate observed in Stevia explants under light exposure might have resulted from high ROS accumulation.

For the selection of transgenic shoots, we found that concurrent visual and antibiotic selection was most suitable for Stevia. Fifty Milligram/liter of kanamycin was insufficient to completely inhibit the regeneration of non-transgenic shoots but higher amounts of kanamycin also reduce overall regeneration rate. The use of GFP for visual selection allowed us to easily identify transgenic shoots without compromising on the regeneration rate and thus maximized transformation rate. Such concurrent antibiotic and visual selection have also been employed for efficient transformation of the rubber tree and the sweet chestnut [36, 37].

The stable integration of *SrDXS1* or *SrKAH* into the genome of transgenic lines was confirmed by genomic PCR and Southern blot analyses. Notably, our genomic Southern blot analysis shows the existence of transgene and its number of integration sites in transgenic Stevia genome. Among transgenic Stevia plants, 46% of the SrDXS1-OE lines and 56% of the SrKAH-OE lines had transgene integrated at a single site (Fig. 4c, d).

Generally, the overexpression of *SrKAH* and *SrDXS1* had increased total SGs content without affecting the proportion of individual SGs in the leaves. However, an exception was observed in SrKAH-OE #4 where its Reb A content was drastically increased by 133–200% but its stevioside content was only increased by 57–71%. As SrUGT76G1 is responsible for the conversion of stevioside to Reb A [10], we measured its transcript levels in the SrKAH-OE lines, but no significant differences were seen amongst them (Additional file 8: Figure S8). Thus, we postulate that SrUGT76G1 in SrKAH-OE #4 could have been affected at the protein level, possibly by enhanced enzymatic activity or greater stability.

For the study with next generation of transgenic Stevia lines, we had difficulty in harvesting viable seeds under our environmental condition. Even though lots of pollen grains were attached to the stigma of the flowers, transgenic and WT seeds that we collected were always empty and non-viable. Nevertheless, by in vitro cutting propagation, we were able to continually obtain clones of the transgenic lines that do not show a reduction in expression levels of the transgene or SG content over time.

Metabolic engineering to increase desirable metabolites in plants can be done through increasing flux towards the relevant pathways by overexpressing rate-limiting enzyme genes in the pathway [38]. We found that the total SGs content was increased by up to 54% in transgenic lines overexpressing *SrDXS1* when compared to vector-only control. In Arabidopsis, upregulation of *DXS* elevated chlorophylls and carotenoids concentration together with GA and abscisic acid content [27]. We also expected that an increase in precursor supply from the MEP pathway in SrDXS1-OE lines might affect the biosynthesis of other downstream metabolites along with SGs. However, the overexpression of *SrDXS1* did not affect levels of chlorophylls, carotenoids and monoterpenes tested. This finding is not unique to Stevia as the overexpression of Arabidopsis *DXS* in spike lavender also led to the higher amount of essential oil but no changes in the chlorophylls and carotenoids levels [39]. The difference in response to elevated *DXS* levels seemed to imply that in plants producing specialized secondary metabolites, excess precursors from the MEP pathway would be diverted to their biosynthesis instead of the biosynthesis of primary metabolites such as the phytohormones and chlorophylls that could have adverse effects on the growth and development of the transgenic plants.

Other common targets for metabolic engineering include the cytochrome P450s as they tend to catalyze rate-limiting and irreversible steps in pathways with high specificity [40]. By overexpressing *SrKAH*, we generated transgenic lines that were up to 88% more abundant in total SGs. The expression levels of *SrKAH* were also found to be positively correlated to the SGs contents in the SrKAH-OE lines. However, steviol but not SGs was previously detected in the leaves of Arabidopsis by heterologous expression of *SrKAH* [30]. This was likely due to the lack of UGTs that could glycosylate steviol. In contrast, steviol remained undetectable in the leaves of the Stevia SrKAH-OE lines, possibly due to rapid glycosylation of newly synthesized steviol by downstream UGTs for sequestration into the vacuoles to avoid its potential toxicity [1]. Overexpression of *SrKAH* in Arabidopsis also led to dwarfism, which is characteristic of plants with reduced GA levels [30]. This was attributed to the diversion of precursors for GA towards steviol biosynthesis. However, our transgenic Stevia plants overexpressing *SrKAH* did not exhibit any observable morphological differences compared to control. SrKAH is an enzyme unique to Stevia that has been evolved for SGs biosynthesis. Therefore, the allocation of precursors for GA or SGs biosynthesis is likely to be under tight control which would allow for the continual accumulation of SGs without affecting normal growth and development of Stevia in response to changes in internal or external factors. However, this mechanism of regulation remains to be investigated.

Comparing between high expressers of SrKAH-OE and SrDXS1-OE lines, the increase in total SGs content in the former was higher than the later. This is likely due to *SrKAH* being situated further down in the SGs biosynthesis pathway allowing its upregulation to have a more direct effect on SGs production. Another possible explanation is that the increased precursors supply from *SrDXS1* upregulation might be diverted to the production of other metabolites unidentified in this study along the many steps in the pathway. There may also be other rate-limiting steps in the pathway restricting the increase in SGs production. Nevertheless, the overexpression of *SrDXS1* increased SGs levels without any obvious unintended effects. We postulate that SGs content could further be enhanced by the co-expression of *SrKAH* and *SrDXS1.* The elevated SrKAH activity would help divert the greater amount of precursors resulting from *SrDXS1* overexpression towards SGs biosynthesis more efficiently, having a push and pull effect [41, 42]. It is recognized that among the two most abundant SGs present in Stevia leaves, Reb A has a sweeter and more pleasant taste profile than stevioside [43]. Hence, it may also be desirable to target the UGTs in the future to engineer Stevia with higher Reb A to stevioside ratio.

## Conclusions

We established an effective method for Stevia transformation demonstrated by the SrDXS1-OE and SrKAH-OE lines. This will serve as an important tool for further overexpression or knockdown studies of newly identified genes from Stevia RNA-seq database. Furthermore, it will also facilitate metabolic engineering of Stevia with greatly enhanced total SGs content and more pleasant tasting SGs including the minor SGs, Reb D and Reb M.

## Methods

### Plant materials and growth condition

*Stevia rebaudiana* Bertoni were propagated and maintained in vitro by cutting and transferring apicals onto fresh RM containing Murashige & Skoog (MS) medium with 6.5 g/L agar and 0.5 mg/L of IAA every 3–4 weeks. The in vitro plants were kept in a LD (16 h L/8 h D) plant growth chamber maintained at 25 °C. After rooting, they were transferred to potting soil mixed with sand and covered for 1 week with a transparent plastic dome for hardening.

### Stevia tissue culture

The second and third leaves (cut into ~ 5 × 5 mm pieces) from sterile 2–3 week-old in vitro propagated plants were used as the explants source for Stevia tissue culture and transformation. Forty pieces of explants were incubated on MS media with six different combinations (Conditions A-F, Table 1) of plant growth regulators under continuous darkness unless otherwise specified. Explants placed on CIM for 3 weeks were assessed for calli formation rates and transferred onto SIM for another 3 weeks to evaluate the percentage of explants with regenerated shoots. One-way analysis of variance (ANOVA) was used to evaluate for differences in the callus formation and regeneration rates between the Conditions [44].

### Functional complementation assay for *SrDXSs* in *Escherichia coli* mutant

*SrDXS1*, *SrDXS2*, *SrDXS3,* and *SrDXS4* amplified from Stevia cDNA using primers listed in Additional file 9: Table S1 were cloned into the pDONR221 and followed by recombination into the pDEST17 using Gateway cloning technology (Invitrogen). The resulting pDEST17-SrDXS constructs were transformed into an *E. coli dxs*^−^ strain defective in DXS activity. For complementation assay, the transformed cells were streaked out on Luria-Bertani (LB) agar plates with 1 mM of mevalonate (MVA) or without MVA and incubated overnight at 37 °C. *AtDXS1* and pDEST17 transformed into the *E. coli dxs*^−^ strain were used as positive and negative controls, respectively.

### Subcellular localization of SrDXS1 and SrKAH

*SrDXS1* and *SrKAH* in the pDONR221 entry clone were transferred into the destination vector pBA-DC-YFP [12] using LR clonase (Invitrogen) and the resulting C-terminal YFP-tagged constructs were transformed into the *Agrobacterium* strain GV3101. The *Agrobacterium* suspension was infiltrated into the leaves of 4-week-old *N. benthamiana* plants and incubated at 24 °C under LD photoperiod for 3 days before excision and mounting on slides for observation under a CLSM (Carl Zeiss LSM 5 Exciter, Germany). Argon laser at 514 nm and 458 nm were used to excite YFP and CFP, respectively. The bandpass were set at 530–600 nm for YFP and 475–525 nm for CFP while the long pass was set at 650 nm. Image processing was done on LSM Image Browser.

### Vector construction for Stevia transformation

The full-length ORFs of *SrDXS1* (accession number: KT276229) [12] and *SrKAH* (accession number, EU722415) [29, 30] were PCR-amplified from cDNA derived from Stevia leaves using primers listed in Additional file 9: Table S1. PCR products were cloned into pK7WG2D using Gateway technology (Invitrogen) to generate pK7WG2D-SrDXS1 and pK7WG2D-SrKAH. All clones were confirmed by sequencing.

### Stevia transformation

Vector constructs used were transformed into the *Agrobacterium* strain AGL2. For co-cultivation, *Agrobacterium* at log phase was pelleted and resuspended in MS supplemented with 100 μM of acetosyringone to OD_600_ of 0.4–0.6. The explants were incubated with the *Agrobacterium* suspension for 30 min with occasional gentle shaking and then placed on CCM (0.25 mg/L 2,4-D + 100 μM acetosyringone) at 22 °C for 3 days in the dark. Following co-cultivation, the explants were washed twice with sterile deionized H_2_O and once in MS media supplemented with 300 mg/L cefotaxime by vigorous shaking before soaking in MS media with cefotaxime for another 20 min. The washed explants were placed on CIM (1 mg/L BA + 0.5 mg/L IAA + 125 mg/L cefotaxime + 50 mg/L kanamycin) for the next 3–4 weeks at 25 °C in the dark for callus induction. The calli were screened under a fluorescence stereomicroscope Leica MZ 10F equipped with a FITC/GFP filter and illuminated by mercury metal halide lamp. Autofluorescence from chlorophyll was not filtered out. Images were captured using a Nikon DXM 1200F camera. Calli showing GFP spots were transferred to SIM (2 mg/L BA + 0.25 mg/L IAA + 125 mg/L cefotaxime + 50 mg/L kanamycin) and subcultured every 3–4 weeks. Regenerated shoots from calli emitting GFP signals were transferred onto RM supplemented with 125 mg/L of cefotaxime. Only shoots on kanamycin containing media with GFP signal present throughout the plant were selected for rooting. Transformation efficiency of this protocol was tested using *Agrobacterium* harboring pK7WG2D in triplicates on 200 pieces of explants. Regenerated transgenic plants with roots formed after approximately 4 weeks on RM were multiplied by cutting the terminal shoot and propagating the lateral shoots. Lines where GFP signals could be detected from leaves of all individuals would then be transferred onto soil in growing trays and covered with a transparent plastic dome for 1 week for hardening.

### Verification of transgenic Stevia plants by genomic PCR and southern blot analysis

Genomic DNA (gDNA) was extracted from approximately 600 mg of Stevia leaves using cetyltrimethylammonium bromide (CTAB)-based extraction method [45]. The final gDNA pellet was washed with ice-cold 75% ethanol and dissolved in water.

PCR amplification was carried out from 100 ng of gDNA extracted from each line of transgenic Stevia to check for the presence of T-DNA using forward primers specific to the CaMV 35S promoter and reverse primers specific to the 3′-end of *SrDXS1* or *SrKAH* (Additional file 9: Table S1).

Southern blot analysis for detection of transgene integrations and number of integration sites was performed using a DIG-labelled probe specific to the full-length *nptII* (Roche)*.* The purity of the synthesized probes was checked by electrophoresis on a 1% agarose gel. gDNAs extracted from the SrDXS1-OE and SrKAH-OE lines were digested with *Hin*dIII and *Xba*I, respectively. After digestion, the fragments were resolved on a 0.8% agarose gel together with DIG-labelled DNA molecular weight marker II (Roche). The agarose gel was treated with 0.2 M HCl followed by denaturation solution (0.5 M NaOH, 1.5 M NaCl) and neutralization solution (1 M Tris-Cl pH 7.4, 1.5 M NaCl) and transferred to a positively charged nylon membrane (Hybond-N+, GE healthcare life sciences) in 20x SSC (3.0 M NaCl, 0.3 M sodium citrate, pH 7.0). After the transfer, UV-crosslinking was carried out using Stratalinker 2400 (Stratagene, USA). Then, DIG-based Southern blot hybridization was performed according to manufacturer’s instructions (Roche). Chemiluminescence from the membrane was acquired with the ChemiDoc Touch Imaging System (Bio-Rad, USA).

### Expression analysis by quantitative real-time PCR (qRT-PCR)

Total RNA was extracted from homogenized Stevia leaves using the TRIzol reagent (Invitrogen) and then treated with deoxyribonuclease I (DNase I; Roche, USA) to avoid possible genomic DNA contamination. Total RNA concentration was measured using a Nanodrop spectrophotometer, ND-1000 (Thermo Fisher Scientific, USA). One μg of total RNA was used for cDNA synthesis with M-MLV Superscript II (Promega, USA).

qRT-PCR was performed using SYBR *Premix Ex Taq* II (Takara, Japan) on the synthesized cDNA. The gene-specific primers are listed in Additional file 9: Table S1. The expression levels were quantified on Applied Biosystems (USA) 7900HT fast real-time PCR system. Stevia *actin* gene was used as an internal control for normalization. Specificity of the amplified PCR products was verified by regular PCR analysis and melting curve analysis on the qRT-PCR system. Biological and technical triplicates were carried out for each experiment.

### Steviol glycosides content analysis by high performance liquid chromatography

To analyze SGs content in the transgenic lines, leaves on the 6th node were harvested from plants grown in the greenhouse for 3 weeks and dried overnight in a 60 °C oven. Sterile water was added at 1 mL per 10 mg of powdered sample and extraction was carried out twice by sonication in a 50 °C water bath for 20 min. The extracts were clarified by centrifugation at 3000 *g* for 15 min and pooled. After filtering through a 0.45 μm filter, 1 mL of the sample was applied to a solid phase extraction (SPE) column C2 (Agilent, USA) and eluted in 1 mL of methanol:acetonitrile (50,50, *v*/v). Eluted samples were analyzed on Shidmadzu Nexera X2 ultra-high performance liquid chromatography (UHPLC) system as described previously [12].

### Chlorophylls and total carotenoids analysis

To analyze the chlorophylls and total carotenoids content in the transgenic lines, 200 mg of leaves homogenized in liquid nitrogen was extracted twice with 2 ml of 100% methanol. Extraction was carried out at room temperature for 1 h in the dark with constant shaking. Methanol fraction from both extracts was pooled and diluted 5 folds before their absorbance values at wavelengths 666 nm, 653 nm and 470 nm were determined using an Infinite M2000 microplate reader (Tecan, Switzerland). The relative amount of chlorophyll *a*, chlorophyll *b* and total carotenoids were calculated from their absorbance values using previously reported formula [46].

### Monoterpene content analysis by GC-MS

Leaves harvested from the 4th and 5th nodes of Stevia plants grown in the greenhouse for 3 weeks were homogenized in liquid nitrogen. Three hundred fifty milligrams of leaf powder was extracted with 350 μL of ethyl acetate containing 20 μg/mL of camphor (Sigma-Aldrich) as an internal standard. After 3 h incubation at room temperature with constant shaking, the ethyl acetate fraction was transferred into a new tube and treated with anhydrous Na_2_SO_4_. The treated extracts were then filtered through a 0.45 μm nylon centrifuge tube (Corning, USA). The GC-MS analysis was performed on Agilent 7890A GC (Agilent Technologies, USA) system as described previously [12].

## Additional files

Additional file 1: **Figure S1.** Representative phenotypes of callus on callus induction media. **a** Calli induced on media containing 1 mg/L BA and 1 mg/L NAA after 6 weeks. **b** Calli and shoot regenerated on media containing 1 mg/L BA and 1 mg/L IAA after 6 weeks. **c** Leaf explants placed for 1 month on media with 1 mg/L BA and 1 mg/L IAA either under 16 h L/8 h D photoperiod (upper panel) or under continuous darkness (lower panel). Scale bar = 1 cm. (PDF 214 kb)

Additional file 2: **Figure S2.** Representative phenotypes of the regenerated shoots. **a** Unhealthy looking regenerated shoots with watery and translucent appearance and slight browning. **b and c** Healthy looking callus with few shoots typical of regenerated shoots under Condition E (**b**) and many regenerated shoots typical of Condition F (**c**). Scale bar = 0.5 cm. (PDF 157 kb)

Additional file 3: **Figure S3.** Immunoblot analyses showing GFP expression in transgenic lines. Total leaf protein was extracted from, SrDXS1-OE, SrKAH-OE and WT lines and probed with α-GFP antibody. Lower panel shows blot after staining with coomassie blue. Extra panel below coomassie blue stained blot shows GFP expression in the SrDXS1-OE lines #7–13 with increased amount of sample loaded and longer exposure time. (PDF 639 kb)

Additional file 4: **Figure S4.** Representative chromatograms from UHPLC analysis of steviol glycosides. **a** Chromatogram of leaf extract from SrDXS-OE #5 compared to that of the Wild type (WT) and standard sample mixture (Standard) of nine steviol glycosides (Rebaudioside D, Rebaudioside A, Stevioside, Rebaudioside F, Rebaudioside C, Dulcoside A, Rubusoside, Rebaudioside B, Steviolbioside) as indicated on the diagram. **b** Chromatogram of leaf extract from SrKAH-OE #1 aligned with that of WT and Standard. (PDF 172 kb)

Additional file 5: **Figure S5.** Relative content of Reb C and dulcoside A detected from the dried leaves of transgenic Stevia. **a** Amount of Reb C and Dulcoside A relative to wild type (WT) control line in the *SrDXS1* overexpressing lines (SrDXS1-OE). **b** Relative abundance of Reb C and Dulcoside A in the *SrKAH* overexpression lines (SrKAH-OE) relative to the wild type (WT) control line. All SGs were detected using HPLC at wavelength of 210 nm. Statistical analysis were carried out using Student’s *t*-test relative to wild-type (WT) (*n* = 5, * *p* < 0.05, ** *p* < 0.01). Data are presented as mean ± SE. (PDF 183 kb)

Additional file 6: **Figure S6.** Total content of stevioside and Reb A in SrDXS1-OE line #9. Measurements were made on fresh leaves pooled from five individuals. (PDF 176 kb)

Additional file 7: **Figure S7.** Monoterpenes extracted from Stevia plants overexpressing *SrDXS1* (SrDXS1-OE). **a-c** α-pinene (**a**), β-pinene (**b**), and linalool (**c**), extracted from the leaves. All measurements are expressed as mean ± SE and statistical analysis was carried out using Student’s *t*-test (*n* = 5). (PDF 254 kb)

Additional file 8: **Figure S8.** Transcript levels of *SrUGT76G1* in SrKAH-overexpression lines (SrKAH-OE). The values are expressed as mean ± SE (*n* = 3). Student’s *t*-test was used for the analysis of statistical significance. (PDF 175 kb)

Additional file 9: **Table S1.** List of primers used in study. (PDF 182 kb)

## Abbreviations

BA: 6-benzylaminopurine
CaMV: Cauliflower mosaic virus
CCM: Co-cultivation media
CFP: Cyan fluorescent protein
CIM: Callus induction media
CLSM: Confocal laser scanning microscope
DIG: Digoxygenin
DMAPP: Dimethylallyl pyrophosphate
DXP: 1-deoxy-d-xylulose-5-phosphate
ER: Endoplasmic reticulum
GC-MS: Gas chromatography-mass spectrometry
GFP: Green fluorescent protein
GGPP: Geranylgeranyl pyrophosphate
GUS: β-glucuronidase
IPP: Isopentenyl pyrophosphate
LD: Long day
MEP: Methylerythritol phosphate
MS: Murashige & Skoog
MVA: Mevalonate
NAA: 1-naphthaleneacetic acid
nptII: Neomycin phosphotransferase
ORF: Open reading frame
Reb A: Rebaudioside A
RM: Rooting media
ROS: Reactive oxygen species
SGs: Steviol glycosides
SIM: Shoot induction media
SrDXS1: Stevia 1-deoxy-d-xylulose-5-phosphate synthase 1
SrKAH: Stevia kaurenoic acid hydroxylase
UGT: UDP glycosyltransferase
YFP: Yellow fluorescent protein
