# Supplementary figures and images for: Overexpression of SrDXS1 and SrKAH enhances steviol glycosides content in transgenic Stevia plants
Source: BMC Plant Biol. 2019 Jan 3;19:1. doi: 10.1186/s12870-018-1600-2 (PMC6318952; doi:10.1186/s12870-018-1600-2)

**Figure S1**

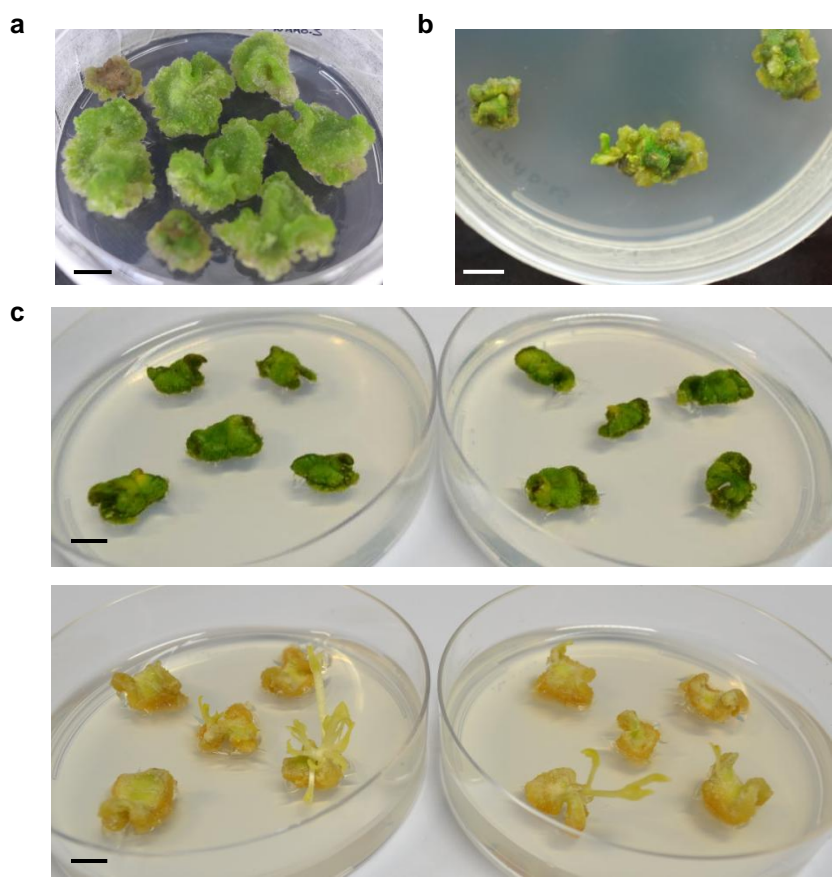

Supplement: Supplementary file 1 — Figure S1. Representative phenotypes of callus on callus induction media. a Calli induced on media containing 1 mg/L BA and 1 mg/L NAA after 6 weeks. b Calli and shoot regenerated on media containing 1 mg/L BA and 1 mg/L IAA after 6 weeks. c Leaf explants placed for 1 month on media with 1 mg/L BA and 1 mg/L IAA either under 16 h L/8 h D photoperiod (upper panel) or under continuous darkness (lower panel). Scale bar = 1 cm. (PDF 214 kb) [file 12870_2018_1600_MOESM1_ESM.pdf]

**Figure S2**

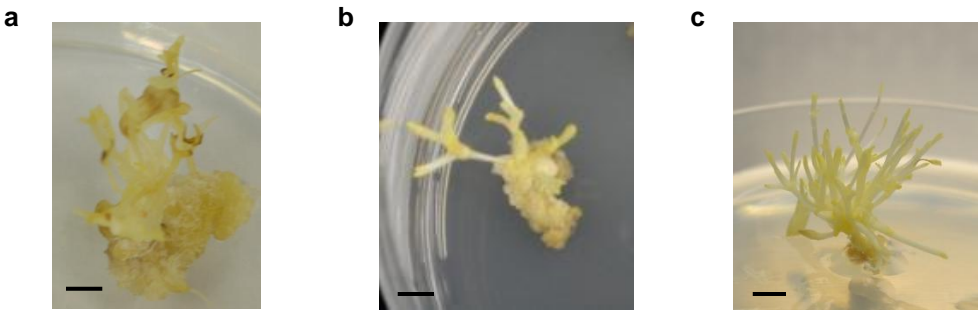

Supplement: Supplementary file 2 — Figure S2. Representative phenotypes of the regenerated shoots. a Unhealthy looking regenerated shoots with watery and translucent appearance and slight browning. b and c Healthy looking callus with few shoots typical of regenerated shoots under Condition E (b) and many regenerated shoots typical of Condition F (c). Scale bar = 0.5 cm. (PDF 157 kb) [file 12870_2018_1600_MOESM2_ESM.pdf]

Figure S3

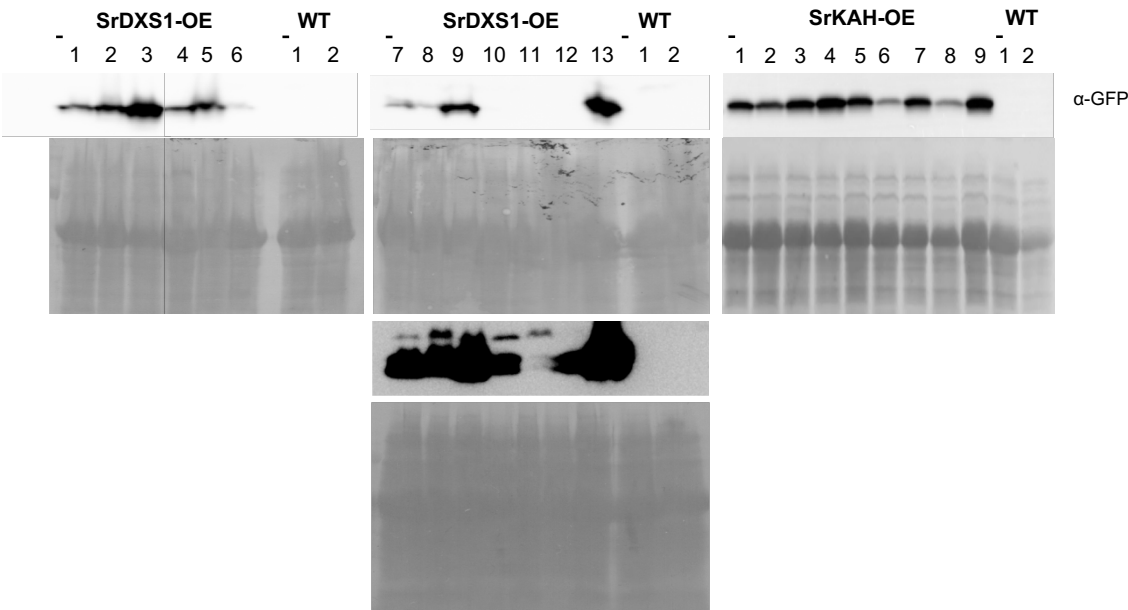

Supplement: Supplementary file 3 — Figure S3. Immunoblot analyses showing GFP expression in transgenic lines. Total leaf protein was extracted from, SrDXS1-OE, SrKAH-OE and WT lines and probed with α-GFP antibody. Lower panel shows blot after staining with coomassie blue. Extra panel below coomassie blue stained blot shows GFP expression in the SrDXS1-OE lines #7–13 with increased amount of sample loaded and longer exposure time. (PDF 639 kb) [file 12870_2018_1600_MOESM3_ESM.pdf]

Figure S4

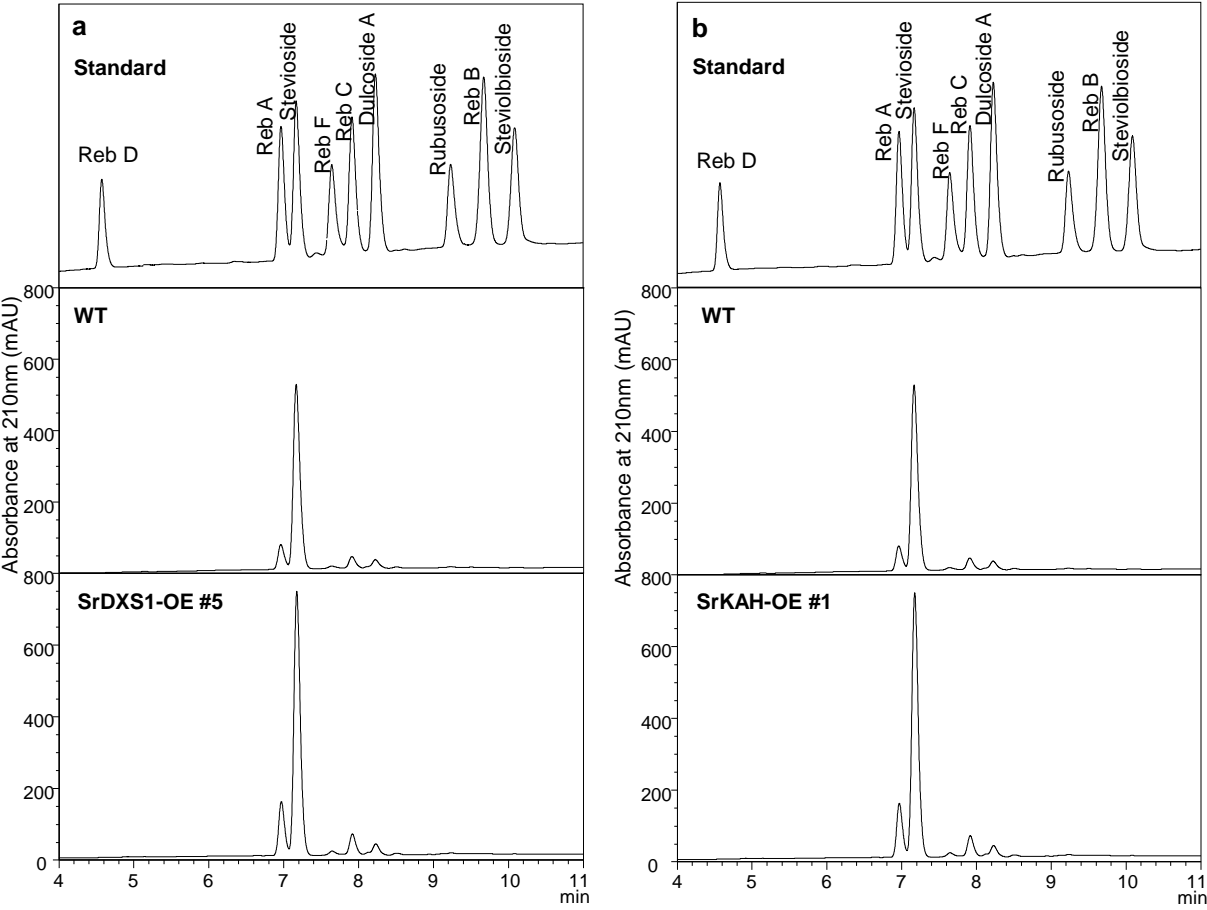

Supplement: Supplementary file 4 — Figure S4. Representative chromatograms from UHPLC analysis of steviol glycosides. a Chromatogram of leaf extract from SrDXS-OE #5 compared to that of the Wild type (WT) and standard sample mixture (Standard) of nine steviol glycosides (Rebaudioside D, Rebaudioside A, Stevioside, Rebaudioside F, Rebaudioside C, Dulcoside A, Rubusoside, Rebaudioside B, Steviolbioside) as indicated on the diagram. b Chromatogram of leaf extract from SrKAH-OE #1 aligned with that of WT and Standard. (PDF 172 kb) [file 12870_2018_1600_MOESM4_ESM.pdf]

Figure S5

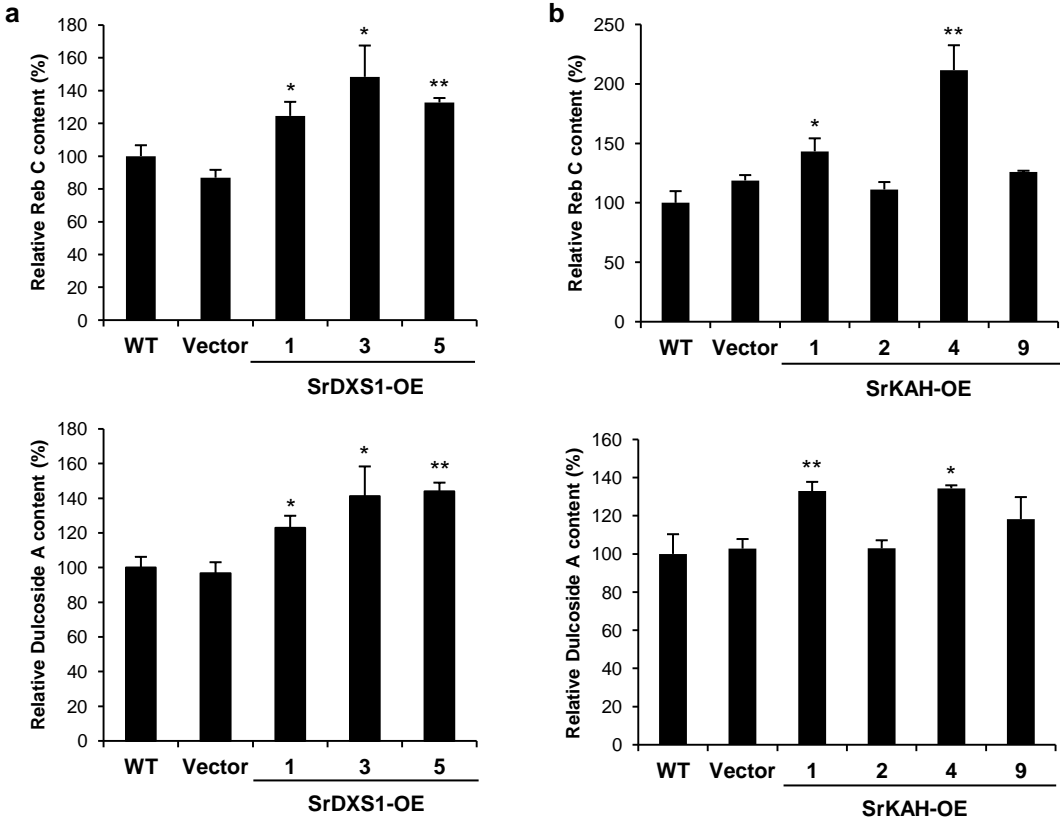

Supplement: Supplementary file 5 — Figure S5. Relative content of Reb C and dulcoside A detected from the dried leaves of transgenic Stevia. a Amount of Reb C and Dulcoside A relative to wild type (WT) control line in the SrDXS1 overexpressing lines (SrDXS1-OE). b Relative abundance of Reb C and Dulcoside A in the SrKAH overexpression lines (SrKAH-OE) relative to the wild type (WT) control line. All SGs were detected using HPLC at wavelength of 210 nm. Statistical analysis were carried out using Student’s t-test relative to wild-type (WT) (n = 5, * p < 0.05, ** p < 0.01). Data are presented as mean ± SE. (PDF 183 kb) [file 12870_2018_1600_MOESM5_ESM.pdf]

Figure S6

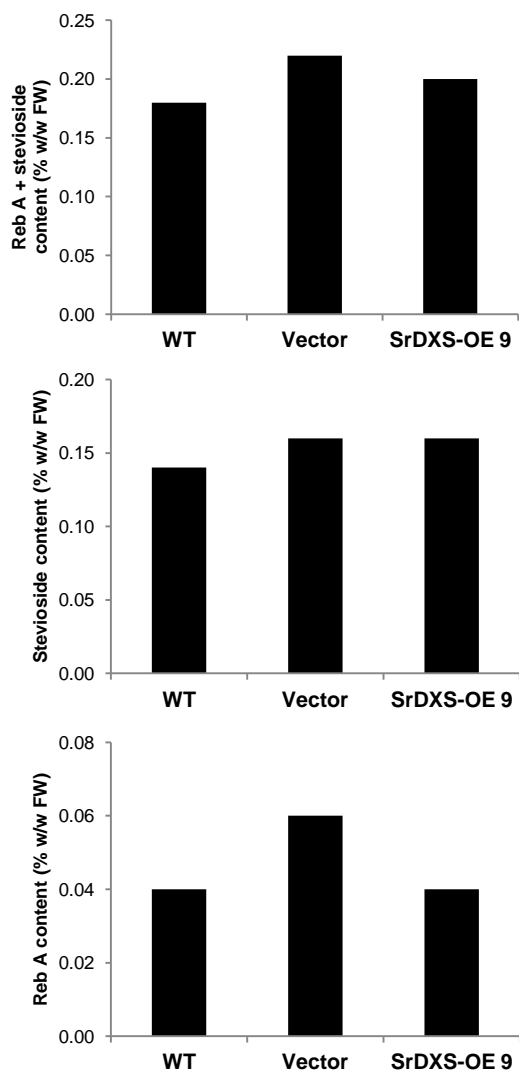

Supplement: Supplementary file 6 — Figure S6. Total content of stevioside and Reb A in SrDXS1-OE line #9. Measurements were made on fresh leaves pooled from five individuals. (PDF 176 kb) [file 12870_2018_1600_MOESM6_ESM.pdf]

**Figure S7**

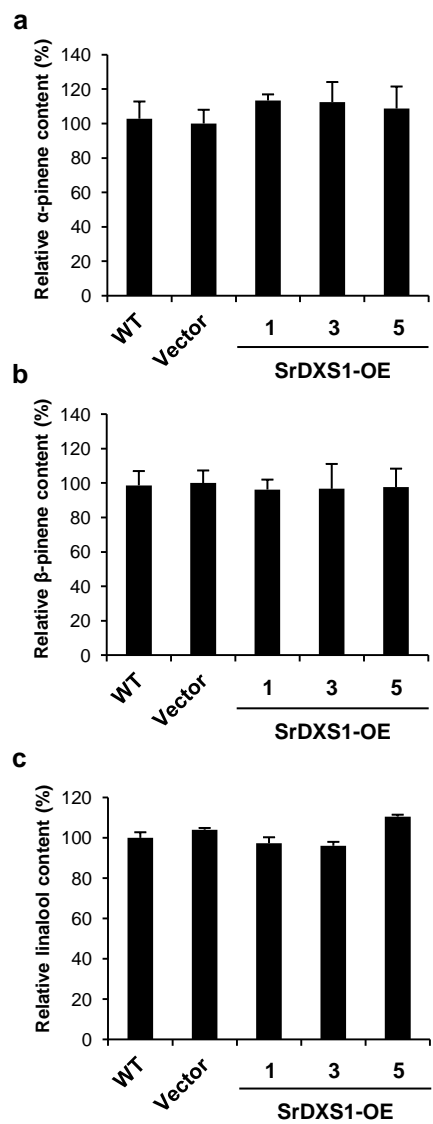

Supplement: Supplementary file 7 — Figure S7. Monoterpenes extracted from Stevia plants overexpressing SrDXS1 (SrDXS1-OE). a-c α-pinene (a), β-pinene (b), and linalool (c), extracted from the leaves. All measurements are expressed as mean ± SE and statistical analysis was carried out using Student’s t-test (n = 5). (PDF 254 kb) [file 12870_2018_1600_MOESM7_ESM.pdf]

**Figure S8**

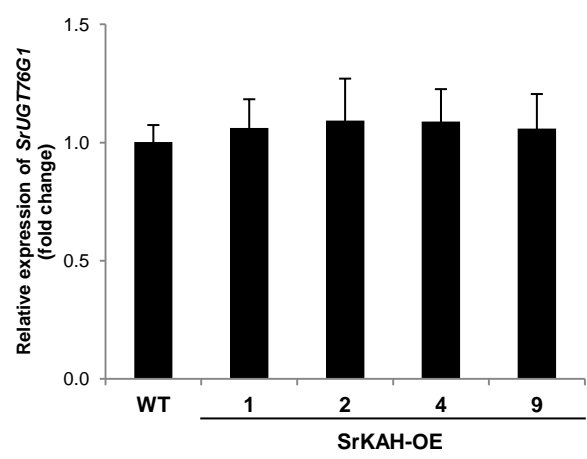

Supplement: Supplementary file 8 — Figure S8. Transcript levels of SrUGT76G1 in SrKAH-overexpression lines (SrKAH-OE). The values are expressed as mean ± SE (n = 3). Student’s t-test was used for the analysis of statistical significance. (PDF 175 kb) [file 12870_2018_1600_MOESM8_ESM.pdf]
