# Supplementary material for: Overexpression of SrDXS1 and SrKAH enhances steviol glycosides content in transgenic Stevia plants
Source: BMC Plant Biol. 2019 Jan 3;19:1. doi: 10.1186/s12870-018-1600-2 (PMC6318952; doi:10.1186/s12870-018-1600-2)
Supplement: Supplementary file 9 — Table S1. List of primers used in study. (PDF 182 kb) [file 12870_2018_1600_MOESM9_ESM.pdf]

Table S1 List of primers used in study

Name	Forward sequence (F)	Reverse sequence (R)
	**For Gateway cloning**	
SrDXS1	*AAAAAGCAGGCTTC* ATGGCGATTTGTGCCTTTGCATTCCCG	*AGAAAGCTGGGTG* TGACATAACCTCCAGAGCCTCTCGG
SrDXS2	*AAAAAGCAGGCTTC* ATGGCTTTATGTGGTGCTTTGAAGGGTG	*AGAAAGCTGGGTG* TAATACATTGACAGCATGTAGCATCTCCTTGC
SrDXS3	*AAAAAGCAGGCTTC* ATGACTACTGCTTCTGCACATTGTTCTTTGG	*AGAAAGCTGGGTG* ACACATCAAAAGAAGAGCTTCACGGGTTC
SrDXS4	*AAAAAGCAGGCTTC* ATGGCGGTTGCAGGATCGACCATGAA	*AGAAAGCTGGGTG* CATTATTGATTTGTATTGAAGTGCTTCTTTAGGT
SrKAH	*AAAAAGCAGGCTTC* ATGATTCAA GTTCTAACACCGATCC	*AGAAAGCTGGGTG* TCAAACTTGATGGGGATGAAGACG
SrKAH_P2	*AAAAAGCAGGCTTC* GGAGCAGCTATCAGGATTGAACTA	*AGAAAGCTGGGTG* TCAAACTTGATGGGGATGAAGACG
	**For RT-PCR**	
SrDXS1	GCAACACTGTCGGAGAGAGGTG	CTGTTAACTCCACCACACCAAGAC
SrKAH	GAGCAACTAGAGATATCGAAGACG	CACTCCAGTGTAGCTTCCATCCT
SrUGT76G1	TATTCCCGGTACCATTTCAAGGC	CGGTAGATTGGAAATGCGTTCGTC
SrActin	TCTTGATCTTGCTGGTCGTG	GAGCAAGAACTTGAAACCGC
	**For confirmatory PCR in transgenic lines**	
SrDXS1-OE	TAGAGAGGCCTACGCGGCAGGT	AGAAAGCTGGGTGTGACATAACCTCCAGAGCCTCTCGG
SrKAH-OE	TAGAGAGGCCTACGCGGCAGGT	AGAAAGCTGGGTGTCAAACTTGATGGGGATGAAGACG
	**For Southern blot probes**	
NptII	ATGATTGAACAAGATGGATTGCACGCAG	TCAGAAGAACTCGTCAAGAAGGCGATAG
